# Development of a Deep-Learning Pipeline to Recognize and Characterize Macrophages in Colo-Rectal Liver Metastasis

**DOI:** 10.3390/cancers13133313

**Published:** 2021-07-01

**Authors:** Pierandrea Cancian, Nina Cortese, Matteo Donadon, Marco Di Maio, Cristiana Soldani, Federica Marchesi, Victor Savevski, Marco Domenico Santambrogio, Luca Cerina, Maria Elena Laino, Guido Torzilli, Alberto Mantovani, Luigi Terracciano, Massimo Roncalli, Luca Di Tommaso

**Affiliations:** 1 Artificial Intelligence Center, IRCCS Humanitas Research Hospital, Via Manzoni 56, 20089 Rozzano, Italy; pierandrea.cancian@humanitas.it (P.C.); victor.savevski@humanitas.it (V.S.); mariaelena.laino@humanitas.it (M.E.L.); 2 Department of Pathology, IRCCS Humanitas Research Hospital, Via Manzoni 56, 20089 Rozzano, Italy; marco.dimaio@alumni.hunimed.eu (M.D.M.); luigi.terracciano@hunimed.eu (L.T.); massimo.roncalli@hunimed.eu (M.R.); 3 Department of Electronics, Information and Bioengineering, Politecnico di Milano, 20133 Milan, Italy; marco.santambrogio@polimi.it (M.D.S.); luca.cerina@polimi.it (L.C.); 4 Department of Immunology and Inflammation, IRCCS Humanitas Research Hospital, Via Manzoni 56, 20089 Rozzano, Italy; nina.cortese@humanitasresearch.it (N.C.); alberto.mantovani@humanitasresearch.it (A.M.); 5 Department of Biomedical Science, Humanitas University, Via Rita Levi Montalcini 4, Pieve Emanuele, 20090 Milan, Italy; guido.torzilli@hunimed.eu; 6 Department of Hepatobiliary and General Surgery Humanitas, IRCCS Humanitas Research Hospital, Via Manzoni 56, 20089 Rozzano, Italy; 7 Hepatobiliary Immunopathology Unit, IRCCS Humanitas Research Hospital, Via Manzoni 56, 20089 Rozzano, Italy; cristiana.soldani@humanitas.it; 8 Department of Biotechnology and Translational Medicine, University of Milan, 20133 Milan, Italy; 9 The William Harvey Research Institute, Queen Mary University of London, London EC1M 6BQ, UK

**Keywords:** macrophages, colo-rectal liver metastases, deep learning, artificial intelligence, digital pathology

## Abstract

**Simple Summary:**

We recently proved that in human colorectal cancer, the presence of small or large tumor-associated macrophages (TAMs) is associated with different outcomes. To translate this biological data into a robust clinical marker means to identify in a single slide all TAMs, hundreds of cells, and then evaluate the area of each of them, a task unfeasible in the routine pathology workout. With the aim to develop a deep-learning pipeline to tackle this challenge, we selected, trained and tested three different approaches. The deep-learning pipeline based on the DeepLab-v3 architecture and semantic segmentation technique warrants the separation of TAMs from the background and the identification of single TAMs: this will easily allow the evaluation of their area.

**Abstract:**

Quantitative analysis of Tumor Microenvironment (TME) provides prognostic and predictive information in several human cancers but, with few exceptions, it is not performed in daily clinical practice since it is extremely time-consuming. We recently showed that the morphology of Tumor Associated Macrophages (TAMs) correlates with outcome in patients with Colo-Rectal Liver Metastases (CLM). However, as for other TME components, recognizing and characterizing hundreds of TAMs in a single histopathological slide is unfeasible. To fasten this process, we explored a deep-learning based solution. We tested three Convolutional Neural Networks (CNNs), namely UNet, SegNet and DeepLab-v3, with three different segmentation strategies, semantic segmentation, pixel penalties and instance segmentation. The different experiments are compared according to the Intersection over Union (IoU), a metric describing the similarity between what CNN predicts as TAM and the ground truth, and the Symmetric Best Dice (SBD), which indicates the ability of CNN to separate different TAMs. UNet and SegNet showed intrinsic limitations in discriminating single TAMs (highest SBD 61.34±2.21), whereas DeepLab-v3 accurately recognized TAMs from the background (IoU 89.13±3.85) and separated different TAMs (SBD 79.00±3.72). This deep-learning pipeline to recognize TAMs in digital slides will allow the characterization of TAM-related metrics in the daily clinical practice, allowing the implementation of prognostic tools.

## 1. Introduction

During the last two decades, technological advances have transformed histopathological glass slides into high-resolution digital slides [1]. The availability of digital slides, in turn, has allowed the introduction and application of image analysis methods to histopathology, as previously happened to radiology. Image analysis methods enable recognizing, differentiating, and quantifying images and finally allows the development of Computer-Aided Diagnosis (CAD) tools. Once applied to images, these instruments support the diagnostic process, by highlighting a variety of aspects of interest. In recent years, deep learning has risen to popularity among other image analysis algorithms, due to their superior performance and generalization [2]. These latter models are characterized by an input layer (image data), hidden layers and an output layer (predictions): A representation of human neural architecture known as artificial neural networks [3]. A specific neural network architecture known as Convolutional Neural Network (CNN) is the standard for image recognition [4,5]. CNNs are essentially made by a cascade of filters automatically tuned to extract meaningful information from the input image data. In the field of histopathology, CNNs have already been used for several tasks: from the detection of a simple object as a mitotic figure [6], through the classification of prostate cancer grading [7], to the intriguing potential to pick up, from a simple H/E staining, information regarding the prognosis [8], the response to treatment [9] or even the presence of molecular alterations [10].

Malignant tumors are composed of a heterogeneous population of cancer cells, admixed with a variety of host cells and secreted molecules, namely the Tumor Microenvironment (TME) whose dynamic interactions determine whether the tumor is eradicated or progresses, in a Darwinian-type evolutionary process [11]. Efforts aimed at implementing deep-learning solutions have been convincingly made also in the onco-immunology field, where quantitative analysis of immune cells and TME components has produced relevant results in terms of identification of prognostic biomarkers and better patient profiling [12,13,14,15]. The possibility to adopt a CAD method for the evaluation of T cells has been robustly tested and validated in colo-rectal cancer [16]. As to macrophages, plenty of which populate the tumor microenvironment [17,18,19,20,21,22], there is no clear association with clinical outcomes across cancers [23], possibly due to their profound heterogeneity in terms of polarization, functions, and tissue localization.

We have recently shown that Tumor Associated Macrophages (TAMs) morphology is associated with distinct transcriptomic profiles and clinical outcomes in human Colo-Rectal Liver Metastases (CLM) [22]. In particular, when we separated TAMs according to their area, we observed that small (S) and large (L) TAMs correlated with a 5-year disease-free survival rate of 27.8% and 0.2% respectively (*p* < 0.0001). This was associated with different molecular profiles of small and large populations, particularly in their lipid metabolism and phagocytic repertoire. However, despite the fact that the prognostically negative TAMs can be identified under the microscope, being characterized by larger size, irregular borders and foamy cytoplasm, the assessment and categorization of TAM morphology is unfeasible in the daily clinical practice, since they are admixed with hundreds of other similar macrophages, with distinct transcriptional profile or prognostic values. Overall, their recognition and proper characterization by hand, would result in being time consuming and an impossible task for clinical purposes. Based on these premises, we aimed at developing a deep-learning pipeline able to systematically recognize all TAMs in a CLM specimen. Future studies aimed at evaluating TAM-related features as prognostic or predictive markers would benefit from this digital tool. Once validated in large cohorts, this tool could be integrated in the routine assessment of human CLM and aid in the histopathological report, with consequent translational impact.

## 2. Materials and Methods

### 2.1. Dataset

This study was conducted on the same cohort of patients included in the Donadon et al. [22] study. We included patients aged > 18 yr with histologically proven Colorectal Liver Metastasis that underwent hepatectomy at the Humanitas Clinical and Research Center–Istituto di Ricovero e Cura a Carattere Scientifico between 2005 and 2017. 2 μm-thick consecutive tissue sections were prepared from formalin-fixed and paraffin-embedded tissues, provided by the Pathology Department of the Humanitas Clinical and Research Center, and processed for immunohistochemistry. The sections were then incubated with a primary antibody anti-human CD163 (Leica Biosystems, 10D6 clone, diluted 1:200) for 1 h at room temperature, followed by incubation with the detection system EnVision+System HRP-labeled anti-mouse (Dako). The tissue slides were then digitized using a computer-aided slide scanner (Olympus VS120 DotSlide). Non-contiguous, non-overlapping microscopic areas in the peritumoral area, including CD163+ cells, were considered. Criteria to select the ROIs [22] included: localization at least 1 mm far from the tumor border, absence of necrosis and absence of large vessels. For each patient we extracted three 1920×1080 images, for a total of 303 images.

Only patients with partial response to preoperative therapy or stable disease were included in the study. Patients with the following criteria were excluded from the study: progressive disease, which is the evidence of CLM progression during neoadjuvant systemic therapy; combination of hepatectomy with radiofrequency or microwave ablation; and nonradical hepatectomy, which is the incomplete tumor removal from the liver.

### 2.2. Data Annotation

Data annotation required drawing the contour of every single TAM in every single original histopathological image, a procedure performed under the strict supervision of an expert pathologist (LDT) using an open-source graphic-manipulation software, GIMPv2.10. TAMs were then assigned to the “foreground” class while hepatocytes, cholangiocytes and other normal structures of liver parenchyma were grouped together in the “background” class. At the end of this process original histopathological pictures were coupled with black (background) and white (TAM) images which provide the ground truth, i.e., the solution to the problem.

### 2.3. Data Augmentation

To virtually enlarge the dataset we applied aggressive data augmentation [24,25]. This process applies any of the following transformations to the input data: rotation, scaling, shear, warp, color jitter, and changes to contrast, hue and exposure. In particular, morphological transformations were applied with a random factor in the (0.8, 1.3) interval, while the rotations could happen at any angle.

### 2.4. Deep Learning Models

The deep learning architectures that we considered in this work were the UNet [26], the SegNet [27] and the DeepLab-v3 [28]. Unless otherwise stated all the models use the architecture described in their cited original paper. The DeepLabV3 is a powerful semantic segmentation architecture that leverages a full encoder-decoder and atrous convolutions to capture details at different scales. All the models were trained until the validation loss diverged from the training loss. We used the Adam optimizer [29] with a learning rate of $10^{-4}$, decaying by a factor of 2 when the loss stalled for 15 consecutive iterations. We used the cross entropy loss at the pixel level for all the experiments and, in the case of instance segmentation, we added the discriminative loss function described in the study of Falk et al. [30]. The models were fed randomly extracted 224 × 224 patches at 60× magnification.

### 2.5. Deep Learning Strategies

#### 2.5.1. Semantic Segmentation: Per-Pixel Weights

To improve the ability of our semantic segmentation models to separate single cells, we employed a per-pixel penalty. We assigned different weights to different pixels when computing the loss during training; pixels with a higher weight contributed more to the final loss when misclassified; on the contrary those with lower weight incurred in less penalty. In particular, the pixels lying between TAMs that were closer than 10 pixels were associated with a penalty of about thrice as much, to force the model to recognize the single instance of every cell. We also reduce the penalty for all the pixels on the edge of the cells, to account for imperfect annotations caused by the blurry appearance of the cells. The weights have been determined experimentally, running several trials until the metrics converged to a satisfying result.

#### 2.5.2. Instance Segmentation

We opted for the pixel embedding strategy, modifying a semantic segmentation model following the work by De Brabandere et al. [24] with minor modifications. These authors proposed a new loss function that drives pixels belonging to the same instance in discrete clusters by embedding them in a high dimensional space. The single clusters are then isolated with a standard clustering algorithm at prediction time. In the original work the model’s last convolutional layer is duplicated to produce two outputs, one for conventional semantic segmentation and one n-dimensional embedding space. In our work we found that duplicating the entire decoder branch of a DeepLab-v3 architecture led to the best results.

### 2.6. Metrics

To describe the quality of the segmentation mask, we use two different metrics for each experiment: the Intersection over Union (IoU), as a metric for semantic segmentation quality, and the Symmetric Best Dice (SBD) as a metric for instance segmentation quality. The IoU is a statistic for measuring the similarity between the predicted segmentation mask and the ground truth mask; it is defined as the size of the intersection divided by the size of the union of the masks. With *P* the predicted mask and *T* the ground truth mask, we can express this concept with the formula:

(1) $$\frac{P\cap T}{P\cup T}$$

The SBD quantifies how much the single instance overlapped with the most similar ones. It is an affirmed way of assessing the instance separation quality. To understand the formulation of this statistic we had to start from the concept of Dice Score, defined in a similar fashion to the IoU as:

(2) $$2*\frac{P\cap T}{P+T}$$

The dice score tends to penalize less the worst performing cases than the IoU. Furthermore we could define the Best Dice for two segmentation mask $L^{a}$ and $L^{b}$, with M and N instances respectively as:

(3) $$\mathrm{BD}\left(L^{a},L^{b}\right)=\frac{1}{M}\sum_{i=1}^{M}\max_{1\leq j\leq N}2\frac{\left|L_{i}^{a}\cap L_{j}^{b}\right|}{\left|L_{i}^{a}\right|+\left|L_{j}^{b}\right|}.$$

In words: for each instance *a* in $L^{a}$ find the instance *b* in $L^{b}$ that maximized the Dice Loss, then return the mean of of all the computed partial results. The metric was not commutative with respect to $L^{a}$ and $L^{b}$, as:

(4) $$\mathrm{BD}\left(L^{a},L^{b}\right)\neq \mathrm{BD}\left(L^{b},L^{a}\right).$$

The SBD dealt with this by taking the minimum of the two. It follows that:

(5) $$\mathrm{SBD}=\min\left(BD(X,Y),BD(Y,X)\right).$$

## 3. Results

### 3.1. Dataset

The cohort study included 303 images. The images were split, at the patient level, in training, validation and test set with the 70%, 15%, 15% of the data respectively. The results reported below were obtained on the test set, which was held out during the experiments.

### 3.2. Deep Learning Models

To describe the quality of the segmentation mask, we used two different metrics for each experiment: the IoU, as a metric for semantic segmentation quality, and the SBD as a metric for instance segmentation quality. The IoU is a statistic for measuring the similarity between the predicted segmentation mask and the ground truth mask. The SBD quantifies how much the single instance overlaps with the most similar ones. It is an affirmed way of assessing the instance separation quality. The performance of the selected models and strategies, quantitatively described through the IoU and the SBD, are shown in Table 1.

**Figure 1:**
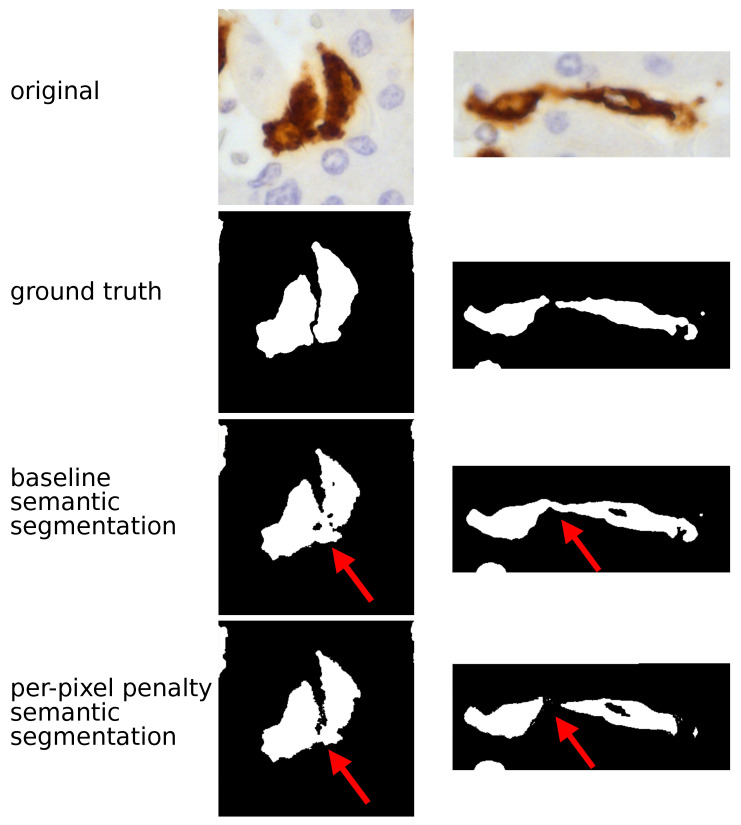
TAM segmentation: semantic approach. The figure compares the histopathological image and the output of CNN model (UNet in these examples) using a semantic approach. In the original image TAMs are stained in brown by CD163 immunostaining and the surrounding hepatocytes (the background) are counterstained in blue; (nuclei darker than cytoplasm); in CNN output TAMs are returned in white and the background in black. The semantic baseline analysis generated an optimal separation between TAMs and the background but was not able to separate adjacent TAMs, as highlighted by red arrows. Adopting the semantic penalty approach fixed part of this specific problem: the two TAMs shown on the right are properly characterized by this solution.

Briefly, the UNet and the SegNet were used at the beginning of the study to establish a baseline, in keeping with previous studies [3,30]. They showed high IoU (82.34±2.59 and 82.13±1.59, respectively) and low SBD (39.87±2.90 and 40.59±4.80, respectively) values. As shown in Figure 1, the baseline semantic segmentation was able to separate TAMs from the background but inadequate to separate adjacent TAMs.

**Figure 2:**
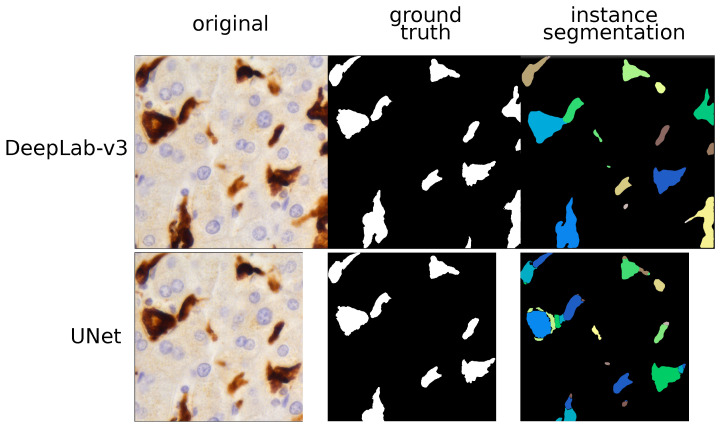
TAM segmentation: instance approach. This figure compares the histopathological image and the outputs given by two models (UNet and DeepLab-v3) using instance segmentation. Different predicted instances are color-coded and we can see that the UNet over-segments most of the TAMs. By contrast, the DeepLab-v3 model properly labelled each TAM as a single instance and did not produce any kind of artifacts.

To overcome this problem, we decided to try a different strategy. Without changing the UNet and the SegNet architecture, we assigned different weights to different pixels contained in regions between one TAM and another. Both UNet and SegNet showed an increase of SBD (61.34±2.21 and 53.90±3.47 respectively) and a decrease of IoU (59.62±3.15 and 54.43±3.87 for UNet and SegNet respectively). Thus, the per-pixel penalty semantic segmentation approach improved the models ability to discern different TAMs, but it was not sufficient to achieve a perfect separation, as shown in Figure 1. Considering these limits, we decided to shift from semantic to instance segmentation. Semantic segmentation assigns to each pixel a class but it considers multiple objects of the same class as a single entity. By contrast, instance segmentation warrants identifying not only the class but also the individual object (instance) each pixel belongs to. In addition to UNet and SegNet we also introduced a more powerful architecture, the DeepLab-v3. For UNet and SegNet, the metrics generated with the instance technique largely overlapped those of the semantic approach. By contrast, the DeepLab-v3 model with instance segmentation showed the highest values for both IoU (89.13±3.85) and, in particular, for SBD (79±3.72). This model allowed the proper identification of adjacent TAMs, as shown in Figure 2.

## 4. Discussion

The goal of this study was the development of a deep-learning pipeline for single TAM segmentation in digital slides of human liver parenchyma next to the colo-rectal metastasis. Segmentation is a computer vision task that involves the identification at the pixel level of particular structures, in this case the TAM. To this aim, we selected, trained and tested three different CNN models (the Unet [26], the SegNet [27] and the DeepLab-v3 [28] with a MobileNet-v2 [31] backbone) and three different strategies (baseline, per pixel weights and instance segmentation).

In the baseline experiments, UNet and SegNet showed a good ability to properly identify the pixels belonging to TAMs. At the same time, low SBD values suggests that these CNN models are unable to discern TAMs that lie close to each other. Taking into consideration that the IoU and SBD have very similar underlying mechanics, the divergent results suggest that these CNN models work properly only in trivial cases, blindly assigning dark patches to the foreground. Most likely, the models were not able to create meaningful high-level features describing the cells as a whole and only leverage low level features such as color and brightness.

Considering the limits raised by the baseline analysis, without changing the UNet and the SegNet architecture, we decided to work towards improving the ability of the models in separating different TAMs. To this aim, we adopted the strategy of assigning a penalty to the misclassified pixels in between TAMs, an approach that yielded promising results in the first UNet publication [26]. As expected, we observed an important increase in terms of SBD for both models, which translated into better results on adjacent TAMs. By contrast, the models showed very prominent artifacts in the segmentation mask. In addition, the penalty strategy had non-trivial overhead in terms of development time. Computing the weights of the ground truth images is time consuming and requires considerable tweaking of the parameters, such as finding an effective value for the penalty or deciding which pixels should be penalized. A further aspect to be taken into account is that imposing the background label in between foreground objects is not a natural way to solve the problem, but rather a workaround to coerce the model into behaving as we desire. We thus considered the possibility that semantic segmentation per se is not the most appropriate approach for our task. Semantic technique treats multiple objects of the same category as a single entity: its goal is to recognize the correct class for each pixel, not to isolate each instance belonging to the same class. This led to the last strategy, explicit instance segmentation.

Interestingly, for both UNet and SegNet, the metrics generated with the instance technique largely overlapped those of the semantic segmentation strategy, confirming the intrinsic limitations of these models to separate TAMs. By contrast, the DeepLab-v3 model with instance segmentation allowed the proper identification of adjacent TAMs.

## 5. Conclusions

A variety of factors within the TME, in particular immune components, have been shown to robustly associate with clinical outcome and therapeutic responses [12,13,14,32]. Despite this, clinically feasible methods to quantitatively and reproducibly evaluate key immune elements are still missing [33]. Notable exception is the Immunoscore in human colo-rectal cancer, a robust prognostic scoring system [12,15] evaluated on digital slides using a CAD tool [16] and recently introduced among the “Essential Criteria” in WHO classification of digestive system tumors [34]. Most of the remaining immune variables can be evaluated only with time-consuming, poorly-reproducible methods which make them unsuitable for practical purposes.

The pipeline that we presented here was developed to overcome limitations due to identification of macrophages in liver parenchyma. Progressive digitalization of histopathology slides and development of deep learning models allowed us to execute this process faster. Indeed, deep-learning solutions can automate and significantly speed-up time-consuming procedures, including searching for a specific cellular type on digital histopathological slides [35]. Altogether these elements allowed the model to investigate at the deepest level the heterogeneity of TAM morphology (avoiding over-segmentation of larger cells) and the complexity of their spatial relationships (avoiding grouping of adjacent cells). Open questions remain on the possibility to analyze Regions of Interest (ROIs), compared to the whole section and to allow the operator or the program to select the Region of Interest (ROI). The most effective strategy could be obtained by a balanced integration of the manual and automatic approaches. In the near future, this pipeline will be tested as a CAD tool to perform quality check over manual annotation, to distinguish, for instance, S- from L-TAM. Other quantitative applications are under study. The ultimate output would be to introduce this pipeline in the routine workout of a pathological report, in order to complement and integrate the manual annotation.

## Figures and Tables

**Table 1:** Numerical results in terms of IoU and SBD for the three models (UNet, SegNet and DeepLab-v3) and the three strategies (baseline, per-pixel penalty and instance segmentation).

Model	IoU Mean	IoU StDev	SBD Mean	SBD StDev
Segnet Vanilla	82.1318	1.59	40.5940	4.80
Unet Vanilla	82.3440	2.59	39.8712	2.90
Segnet Weights	54.4297	3.87	53.9024	3.47
Unet Weights	59.6215	3.15	61.3407	2.21
Deeplab Instance	89.1332	3.85	79.0028	3.72
Unet Instance	75.4104	5.30	37.1577	5.30
Segnet Instance	79.9746	2.15	60.7870	5.75

## Data Availability

The data upon which the models are trained are available under specific request to one of the corresponding authors.

## Abbreviations

The following abbreviations are used in this manuscript:

TME	Tumor Microenvironment
TAM	Tumor Associated Macrophage
CLM	Colo-Rectal Liver Metastases
CNN	Convolutional Neural Network
IoU	Intersection over Union
SBD	Symmetric Best Dice
CAD	Computer-Aided Diagnosis
ROI	Region of Interest
